# Immunocompromised Children and Young Patients Living with Pets: Gaps in Knowledge to Avoid Zoonosis

**DOI:** 10.1155/2023/2151761

**Published:** 2023-05-31

**Authors:** P. Garcia-Sanchez, E. Aguilar-Valero, T. Sainz, C. Calvo, I. Iglesias, D. Bueno, E. Frauca, E. Ramos-Boluda, A. Alcolea-Sanchez, L. García-Guereta, A. Alonso-Melgar, F. Esperón, A. Mendez-Echevarria

**Affiliations:** ^1^Pediatric Emergency Department, La Paz University Hospital Madrid, Madrid, Spain; ^2^Institute for Health Research IdiPAZ, Madrid, Spain; ^3^Pediatric Department, Autonomous University of Madrid, Madrid, Spain; ^4^Pediatric Infectious and Tropical Diseases Department, La Paz University Hospital and Translational Research Network in Pediatric Infectious Diseases (RITIP), Institute for Health Research IdiPAZ, Madrid, Spain; ^5^CIBERINFEC, Carlos III Health Institute, Madrid, Spain; ^6^Pediatric Department, ERN TransplantChild, Autonomous University of Madrid, Madrid, Spain; ^7^Center for Animal Health Research (CISA), INIA, Madrid, Spain; ^8^Pediatric Hemato-Oncology Department, La Paz University Hospital, Madrid, Spain; ^9^Translational Research in Pediatric Oncology, Hematopoietic Transplantation and Cell Therapy, Hospital La Paz Health Research Institute (IdiPAZ), Madrid, Spain; ^10^Pediatric Hepatology Department, Healthcare Working Group, ERN TransplantChild, La Paz University Hospital, Madrid, Spain; ^11^Intestinal Rehabilitation Unit, Pediatric Gastroenterology and Nutrition Unit, ERN TransplantChild, La Paz University Hospital, Madrid, Spain; ^12^Intestinal Rehabilitation Unit, Pediatric Gastroenterology and Nutrition Unit, La Paz University Hospital, Madrid, Spain; ^13^Pediatric Cardiology Department, ERN TransplantChild, La Paz University Hospital, Madrid, Spain; ^14^Pediatric Nephrology Department, ERN TransplantChild, La Paz University Hospital, Madrid, Spain; ^15^Veterinary Faculty, European University of Madrid, Madrid, Spain

## Abstract

**Methods:**

A cross-sectional, observational study was performed in a large tertiary hospital in Madrid, including immunosuppressed patients from different regions of Spain. The participants were asked to complete an online questionnaire.

**Results:**

Two hundred and eighty-four responses were received: 62.3% solid organ transplantation (177/284), 22.8% hematopoietic stem cell transplantation (65/284), and 14.8% inborn errors of immunity (42/284). The median age was 11 years (interquartile range 5.9–15.4), and 55% were boys (156/284). Up to 45% (130/284) of the respondents lived with 201 pets (74% of them dogs and cats). Half of the patients owning dogs or cats did not comply with at least one of the recommendations regarding vaccination, deworming, feeding, and/or veterinarian recommended controls. The poorest findings were related to deworming regimens. Only 42.8% (117/273) of the participants received specific recommendations from their healthcare professionals about companion animals. However, up to 97% of the families considering acquiring a pet did so when the professional did not contraindicate it (31/32), while 72% of the families having pets got rid of their pets when they were advised against animals (8/11).

**Conclusions:**

Pet ownership is frequent among immunocompromised children. They presented risky exposures for acquiring zoonoses, and basic veterinary recommendations were not frequently followed. The opinion of professionals significantly influenced the decision to acquire pets, but less than half of the families received recommendations in this regard.

## 1. Introduction

Pets are known to play an important role in the socio-emotional development of children [1], and contact with animals could have additional beneficial effects on children with chronic medical conditions [1–3]. However, animal contact can also imply risks, especially for immunocompromised children, such as transplanted children or those diagnosed with inborn errors of immunity (IEI) [4]. A variety of zoonoses can be transmitted to humans from their companion animals [5], and immunosuppression can not only increase the risk of acquisition but also the severity of viral, bacterial, and parasitic infections. Opportunistic infections, uncommon in immunocompetent children, are also a risk in these more vulnerable patients [6].

Many families facing the diagnosis of a chronic disease in their children acquire pets in an attempt to provide emotional support and to increase their children's quality of life [7]. However, some data regarding the number of immunosuppressed children who safely keep pets in their household are currently available [4]. In addition, there are considerable biases in the published literature. Cases of zoonosis transmitted from pets to immunocompromised patients have been reported [8–12] although few studies have determined precisely what proportion of human disease is attributable to pets [4, 13].

In this scenario, compliance with specific hygiene and veterinary recommendations for preventing infections is of utmost relevance [5, 14]. Most clinical guidelines for the management of transplant recipients include specific recommendations for immunocompromised patients living with animals. These guidelines stress the need to employ extreme preventive hygiene measures, avoiding, if possible, cleaning the animal's cage/basket/aquarium/terrarium or having direct contact with the animal's feces [5, 14]. Strict veterinary control of these pets should be reinforced via close veterinary surveillance, emphasizing vaccination status (and avoiding live vaccines) and adjusting deworming strategies [4, 5, 14–17]. Avoiding contact with animals during periods of severe immunosuppression is also advised. Certain types of pets, such as young puppies or exotic animals, are not recommended [4, 5, 14]. However, the evidence is scarce and recommendations are mainly based on expert opinions, extrapolation from other immunocompromised settings, and case series [4].

In this context, few institutions provide specific recommendations regarding pets for immunocompromised patients, and many healthcare providers do not systematically screen for pets or offer specific recommendations for their transplanted patients [18, 19]. Therefore, many families of immunocompromised children might not have received proper recommendations regarding zoonosis prevention and their pets' healthcare, leading to low awareness of the risks and potentially increased exposure risk [19].

Although pet ownership is frequent, no studies have addressed the rate of pet ownership among families of immunocompromised children. The aim of this study was to address the prevalence of children and young patients living with animals in a cohort of immunosuppressed pediatric patients and to describe family awareness regarding zoonosis risks, their attitude, and compliance with the recommendations [5, 14–17].

## 2. Patients and Methods

A cross-sectional, observational study was performed at La Paz Pediatric University Hospital, a large tertiary hospital in Madrid, which is a reference hospital for pediatric transplantation and immunocompromised children. The study was led by the Pediatric Infectious Diseases Department, in collaboration with veterinarians from the Animal Health Research Center of the Spanish National Institute of Agricultural and Food Research and Technology. The study was approved by the local Clinical Research Ethics Committee of La Paz University Hospital (PI-4770).

We included patients who have received a solid organ transplantation (SOT), a hematopoietic stem cell transplantation (HSCT), or who have been diagnosed with IEI before the age of 18 years. Patients included were from different regions of Spain, as our hospital is a reference national center for attending transplanted children and patients diagnosed with IEI.

In order to include young immunocompromised patients, we included patients who fulfilled at least one of the following criteria:

- Had received a SOT in the previous 10 years in our hospital
- Had received an HSCT in the last 5 years or in the last 5–10 years if the immune reconstitution was incomplete and/or required immunosuppressive treatment at the time of the study
- Had been diagnosed with genetically confirmed IEI in the previous 10 years.

After identification by managing clinicians, the families of all the patients fulfilling the inclusion criteria were contacted by telephone. For those willing to participate, an online questionnaire was distributed via email using the “Google Forms questionnaire” platform (Supplementary file (available here)). Patients who were 12 years of age and older completed the questionnaire themselves, whereas in the case of children younger than 12, the parents were asked to complete it. Clinical data were obtained by reviewing the patient's medical records. The questionnaire collected the patient's demographic data: the number, type, and characteristics of the animals in the household; the pet's feeding and/or hygiene habits; the pets' veterinary care; and the family awareness regarding veterinary care. Families/patients were asked whether they recalled having received specific recommendations by the healthcare providers before or during their follow-up regarding animal ownership and care.

The minimum requirements to define adequate/inadequate compliance were based on the recommendations for zoonosis prevention included in the guidelines for general owners regarding dog and cat care [16, 17, 20, 21] and guidelines for safe living after transplantation [5, 14, 15]. According to these guidelines, dogs and cats should be brought to the veterinarian at least once a year [16, 17], must comply with the vaccination schedule [20], and should receive intestinal deworming at least every 3 months (even monthly in case of dogs and cats sharing home with immunocompromised individuals) [21]. Unprocessed or raw food should not be offered [5, 14, 15]. Patients should avoid having contact with their animal's feces or cleaning the animal's cage/basket/aquarium/terrarium, and they should avoid acquiring puppies and kittens younger than 6 months and/or exotic animals [5, 14, 15].

Adequate compliance with veterinary recommendations was considered if the owners followed all of the following four recommendations: visiting the veterinarian at least once a year, complying with the vaccination schedule (core-vaccines), avoiding feeding the pet with unprocessed or raw food, and deworming the animal at least every 3 months.

According to the number of measures with adequate compliance, we considered the following:

- Good compliance: adequate compliance with the four items
- Average compliance: noncompliance in one of the items
- Poor compliance: noncompliance in at least two of the items

In addition, compliance with these measures was also analyzed separately.

The statistical analysis was performed using Stata v16.0 (StataCorp LP, College Station, TX, USA) and Prism v.7.0 (GraphPad, Inc., La Jolla, CA, USA). Values were expressed as absolute frequencies and/or percentages; quantitative data were expressed as either a median and interquartile range, minimum-maximum range, or mean and standard deviation, depending on the data distribution. Categorical variables were compared using the chi-squared and Fisher's exact test, and continuous variables were compared with Student's *t*-test or nonparametric tests, as appropriate. A two-sided value of *p* ≤ 0.05 was considered statistically significant.

## 3. Results

A total of 492 surveys were submitted online, and 284 (57.7%) responses were received. The median age of the included patients was 11 years (interquartile range (IQR) 5.9–15.4), with 55% boys (156/284). Up to 62.3% of the included patients were SOT recipients (177/284), 22.8% HSCT recipients (65/284), and 14.8% were patients diagnosed with IEI (42/284). The patients' features are described in Table 1.

When the survey was launched, 45.8% (130/284) of the patients lived with 201 pets, most of them dogs (108) and cats (41) (Table 1). Before transplantation/IEI diagnosis, 32.4% (92/284) of the patients had pets. Therefore, the presence of pets in these households increased by 41.3% after diagnosis/transplantation. Among these new pets, 20.5% (16/78) entailed a high risk according to the guidelines [5, 14]: 14 were puppies/kittens younger than 6 months of age, there was 1 *Litoria* sp. (a native frog from Oceania), and 1 rabbit was infected with *Encephalitozoon cuniculi*.

**Figure 1:**
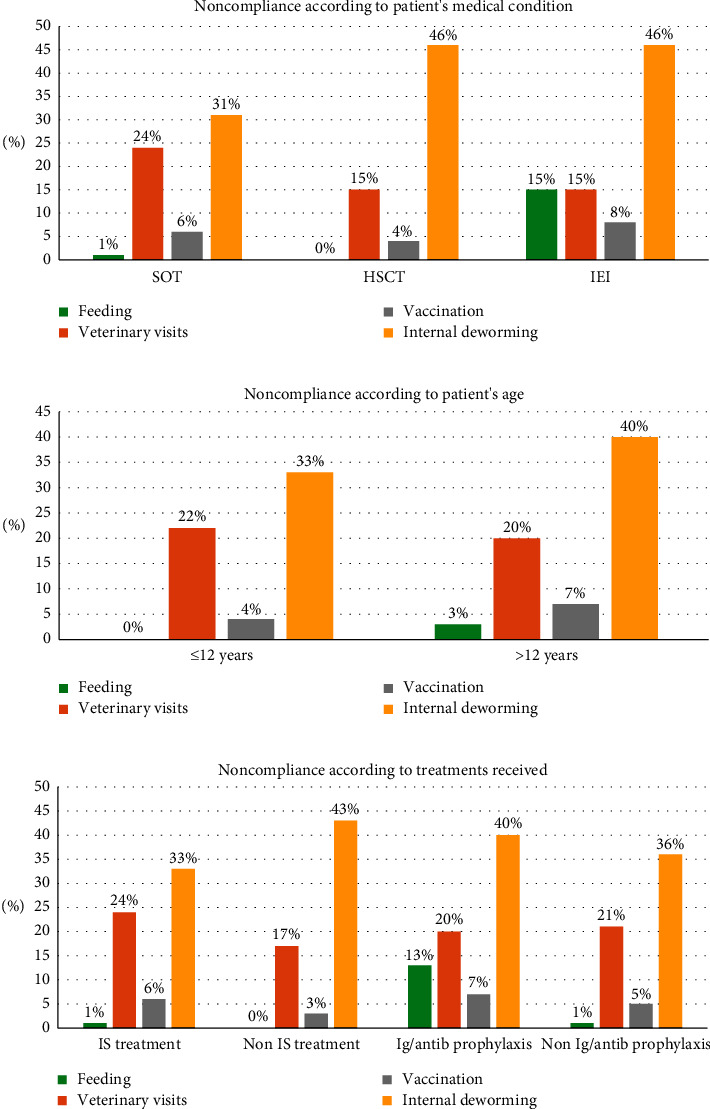
Noncompliance with specific recommendations according to patient's medical condition, patient's age, and treatments received (dogs and cats). ^*∗*^Adequate compliance with veterinary recommendations was considered if the owners followed all the following recommendations: visiting the veterinarian at least once a year, complying with the vaccination schedule, avoiding feeding the pet with unprocessed or raw food, and deworming the animal at least every 3 months.

The patient-pet relationship, food and hygiene habits, and veterinary care are described in Table 2. Among the 130 patients with pets, risk factors for the acquisition of zoonosis were observed in 70% (87/130) of the patients in terms of the pet's age, type of pet, hygiene habits, or failure to comply with the feeding, vaccination, or deworming recommendations. Among the respondents who had dogs and/or cats, up to 50% (53/106) did not comply with at least one of the recommendations regarding vaccination, deworming, feeding, and/or veterinarian recommended controls (Table 2) [5, 14–17].  Table 3 and Figure 1 specify the level of compliance with the various recommendations for dogs and cats according to the patient's medical condition, patient's age, and the treatments received [5, 14–17, 20, 21].

### 3.1. Types of Patients and Pet Ownership

When comparing the various groups of patients, no significant differences were observed in pet ownership according to the patient's medical condition (SOT 44.1%, 78/177; HSCT 50.8%, 33/65; IEI 45.2%, 19/42; *p*=0.6). Pet ownership was more frequent in patients older than 12 years (39%, 58/149) compared to patients younger than 12 (53%, 72/132) (*p*=0.01). Patients who currently had a pet were older (median 4 years, IQR 1.9–11) than those who did not (median 2.6 years, IQR 0.9–8; *p*=0.007). Likewise, the time from transplantation/IEI diagnosis was longer in the patients who lived with pets (median 5.2 years, IQR 2.8–9.6) than in those who did not (median 3.9 years, IQR 2–8.1; *p*=0.003).

### 3.2. Patients' and Families' Perception regarding Pet Ownership

No differences were observed in the number of households with pets when patients were grouped according to their need for immunosuppressive drugs: 43% (79/182) with immunosuppressive drugs and pets vs. 48% (37/77) without immunosuppressive drugs and pets; *p*=0.49. In contrast, those patients who required antibiotic prophylaxis and/or immunoglobulin replacement therapy (IRT) had fewer pets: 30.8% (20/65) vs. 50.5% (110/218); *p*=0.005. However, the pet owners receiving immunosuppressive therapies, antibiotic prophylaxis, and/or IRT often did not properly comply with veterinary recommendations regarding feeding, vaccination, routine veterinary visits, and internal deworming (Table 3). Rates of compliance with these recommendations in dog and cat owners according to their medical condition, age, and type of treatment are shown in Figure 1.

Up to 35.5% (101/284) of the respondents considered pet ownership to be a benefit; however, 41.5% (118/284) thought it posed a risk. Families with pets or those who have ever had one believed that the benefit of having animals outweighed the risks: 58% (81/139) vs. 18% (26/145); *p* < 0.001. Families considering that the benefits of having pets outweigh the risks had older children: 6 years (IQR 1.6–11.4) vs. 2 years (IQR 1–7.2); *p*=0.03. Time since transplantation did not appear to influence the benefit perception, given that no differences were observed when comparing groups in terms of time since diagnosis/transplantation: 5.3 years (IQR 2–9.7) vs. 4 years (IQR 2–8); *p*=0.57. There were no significant differences in the risk perception related to pets among caregivers of patients with different medical conditions (*p*=0.113).

### 3.3. Recommendations Received by Families from Health Professionals

Up to 54.2% (154/284) of the respondents recalled having been asked at some point by their physicians about the presence of pets in their household although only 42.8% (117/273) remembered having received specific recommendations about companion animals. Transplanted patients more frequently received recommendations regarding pets compared with patients with IEI: 47.9% (112/234) vs. 11.9% (5/42); *p* < 0.001. Patients who had undergone HSCT and patients diagnosed with IEI were more often advised against having pets (35.4% (11/31) and 60% (3/5), respectively) compared with professionals attending SOT patients (14.8%; 12/81) (*p*=0.007).

**Figure 2:**
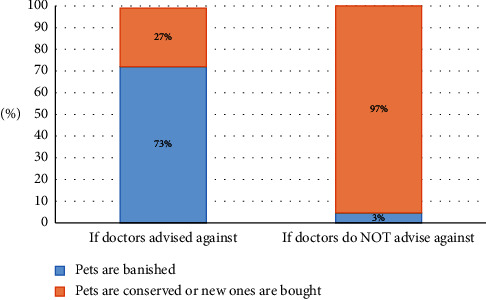
Decisions were made by families who were considering whether to buy or keep a pet at home, based on the recommendations received from their doctors.

Up to 36.8% (43/117) of the families who received specific recommendations about companion animals modified their decision regarding buying or keeping their pet based on the recommendation received. Some 97% (31/32) of those who were considering buying a pet did so when they were not expressly advised against it compared with 27% (3/11) who had been advised not to; likewise, 72.7% (8/11) of the families did not keep their pets when they received recommendations against keeping the animal compared with 3% (1/32) when the presence of pets in the home was not discouraged (*p* < 0.001) (Figure 2).

In summary, our results reveal that pet ownership was frequent (45.8%) and increased after diagnosis/transplantation. Many patients with pets presented some risk factors for the acquisition of zoonoses, and up to 50% of patients with dogs and cats did not comply with at least one of the recommendations regarding vaccination, deworming, feeding, and/or veterinary controls. Only 42.8% of the participants received specific recommendations from their healthcare professionals about companion animals. Despite this, the final decision about pet ownership was influenced by these recommendations in more than one third.

## 4. Discussion

This study is one of the first studies addressing pet ownership among immunocompromised children and young patients. Our results reveal high prevalence of pet ownership (45.8%) in patients from different regions of Spain. Up to 70% of patients with a pet presented some risk factors for the acquisition of zoonoses, and up to 50% of patients with dogs and cats did not comply with at least one of the main veterinary recommendations. On the other hand, less than half of the healthcare professionals provided specific recommendations regarding companion animals. For these reasons, a multidisciplinary one health approach is urgently needed to establish guidelines and recommendations to ensure that our patients can live safely with their pets.

In the European Union and United States, rates of household penetration for pet ownership range from 46% to 68%, with a growth of pet ownership in recent years [22, 23]. Similarly, the number of immunosuppressed pediatric patients has significantly increased in the last decades [24, 25]. Therefore, it is not surprising that the number of immunocompromised patients living with pets is also increasing. Almost half of the patients in our study lived with pets, and the presence of pets in these households increased after their diagnosis/transplantation.

These patients and household members display limited knowledge of pet-associated disease, rarely recall receipt of pet-associated disease information, and report pet ownership practices that are often at odds with established disease prevention recommendations. This reveals the necessity of improving the quality of the recommendations that healthcare providers offer to these families, as well as a need of physicians and veterinarians training in terms of zoonosis prevention. A multidisciplinary one health approach is urgently needed to establish guidelines and recommendations to ensure that our patients can live safely with their pet. In this scenario, promoting veterinarian-physician communication is critical to optimizing the health of both people and animals.

Compliance with basic veterinary care recommendations is mandatory in immunosuppressed patients. However, our results show risky exposures for acquiring zoonoses and low compliance with some of these recommendations. When acquiring a pet, some patients adopted pets that were expressly discouraged, such as puppies/kittens younger than 6 months [14]. Half of the participants owning dogs or cats failed to comply with at least one of the basic veterinary care recommendations, and up to 25% of these patients had poor compliance with the main recommendations. Feeding raw or undercooked food to pets or sharing a bed were practices relatively frequent in our cohort, which entail additional risks and which are risky behaviors that are easy for families to avoid. Many immunocompromised patients collected their animals' feces or cleaned their pet's aquarium, terrarium, cage, or basket. These facts, also reported in previous studies including immunocompromised adults and their pets [26], reflect the low perception of risks among the participating families.

Our results show especially poor compliance data in terms of deworming regimens. The frequency of deworming was lower than the one recommended for the general population (deworm at least every 3 months), and only 11% of the families dewormed their pets monthly, as recommended for cats and dogs sharing homes with young children and/or with immunocompromised individuals [4, 21].

In our study, up to 35.5% of the respondents considered pet ownership to be a benefit. The perception of a benefit that was greater than the risk was observed mostly in patients who already had pets before diagnosis/transplantation and in older patients. Along these lines, most studies have shown that families living with pets believe that their presence in the home is beneficial [7, 27]. Living with animals has demonstrated beneficial effects in children with chronic medical conditions [1–3]; for immunocompromised patients, however, the risk-benefit balance should be discussed individually, as should measures to prevent infections [4]. Although several cases of severe infections in immunocompromised patients transmitted by pets have been documented [8–12], it is unclear to what extent the risk of acquiring infections is increased in this population. Recent studies addressing colonization have reported significant rates of parasites, pathogenic bacteria, or multidrug-resistant microorganisms colonizing pets, and many arthropods, which are potential vectors of infection, are common to humans and pets [9, 28, 29]. In our series, one family reported that their rabbit was diagnosed with an *Encephalitozoon cuniculi* infection once it was living with their child. This intracellular fungus causes severe opportunistic infections in immunocompromised patients [30].

On the other hand, only 54.2% of the respondents to our survey recalled having had the opportunity to discuss pet acquisition/ownership with a healthcare provider and only 42.8% remembered having received specific recommendations. These data are in line with the data from an international survey conducted by our group among healthcare professionals working with transplanted children, in which only 41.2% were found to have actively asked about pet ownership during the anamnesis [19], engendering gaps in knowledge regarding zoonoses that could influence these professionals' clinical practice. In addition, the evidence for the available recommendations regarding specific measurements for patients is generally lacking [4]. As a result, doctors' recommendations are marked by enormous variability and are often based on personal opinions or experiences rather than on scientific evidence. In this context, some of the patients from our cohort were advised against keeping their animals. However, we found no evidence to support this recommendation, indicating once again great variability among practitioners [19].

Given that knowledge of the zoonotic disease risk in pet owners is essential for effective prevention [7], the absence of advice received from their doctors contributes to the low perception of risks among families. Furthermore, our study showed that the recommendation of professionals considerably influenced the family's final decision about the presence of pets in the household. These data should encourage healthcare providers to be aware of the available recommendations and to actively address pet ownership with patients and their families [4] to allow for an informed decision. There is a huge need to improve the diffusion of this knowledge between physicians and veterinarians who attend these children and their pets. Generating evidence to guide clinical practice is urgently needed, especially with the increase in animal therapies in healthcare settings. Although our study has not analyzed the presence of zoonotic infections in immunocompromised children who own pets, it is urgent that this evidence be generated for this group of patients.

This study had some limitations. Families living with pets or who were convinced for or against having pets could have been more prone to engage in the study. In addition, many immunocompromised patients did not complete the survey. This could represent a selection bias. We also think that those families who were more concerned or interested in the matter could have been more prone to answer the survey; therefore, the lack of knowledge about zoonoses and recommendations could be underestimated in this study.

However, this is one of the first studies addressing pet ownership among immunocompromised children, addressing risk perception among families, and indirectly measuring the impact of healthcare advice.

## 5. Conclusions

Pet ownership is common among immunocompromised children and young patients in Spain, and compliance with veterinary recommendations is not good. Half of the participating families did not recall having received recommendations regarding veterinary care or zoonosis prevention from healthcare providers. When provided, recommendations were generally followed. While further evidence is being generated, healthcare professionals need to be aware of the recommendations and become actively involved in discussing pet ownership with the patients/families. Despite significant advances in the One-Health paradigm, there is still much work to be carried out in this scenario involving pets and immunocompromised children. Further analysis will be necessary to quantify risk factors by species and pathogen that can be extrapolated to all types of immunocompromised pet owners (children or not).

## Figures and Tables

**Table 1:** Sociodemographic and disease data of the surveyed population, number, and type of owned pets.

Patient features		Results
Sex		
(i) Female		45.1% (128/284)
(ii) Male		54.9% (156/284)
Median current age		11 years [IQR 5.9–15.4] ≤12 years: 6 years [IQR 3.4–9] >12 years: 16 [IQR 13.6–20]
Median age at diagnosis/transplant		3 years [IQR 1–9.7]
Median time elapsed since diagnosis/transplantation		4.9 years [IQR 2–8.9]
Type of diagnosis:		
(i) Transplant		85.2% (242/284)
SOT		73.1% (177/242)
Liver transplantation		45.8% (81/177)
Kidney transplantation		25.4% (45/177)
Cardiac transplantation		11.9% (21/177)
Multivisceral transplantation		10.7% (19/177)
Intestinal transplantation		6.2% (11/177)
HSCT		26.9% (65/242)
(ii) Inborn errors of immunity		14.8% (42/284)
Current immunosuppressive treatment		64.1% (182/284)
Immunoglobulin replacement therapy		6.3% (18/284)
Antibiotic prophylaxis		20.4% (58/284)

Pet features		Results

Number of pets/patient	0 pet	54.2% (154/284)
	1 pet	28.9% (82/284)
	2 pets	9.2% (26/284)
	3 pets	3.1% (9/284)
	≥4 pets	4.6% (13/284)
Type of pet	Dogs	53.7% (108/201)
	Cats	20.4% (41/201)
	**Birds**	**7% (14/201)** ^*∗*^
	**Turtles/Reptiles**	**6% (12/201)** ^*∗*^
	Fish	4.5% (9/201)
	Rabbits/Hamsters/Guinea pigs	3.9% (8/201)
	**Others**	**4.5% (9/201)** ^*∗*^

SOT: solid organ transplantation; HSCT: hematopoietic stem cell transplantation. ^*∗*^Bold values indicate the type of pets that should be avoided in immunocompromised owners due to their species.

**Table 2:** Data on patients' attitudes, hygiene, feeding, and veterinary care of pets.

Patient-pet relationship	The pet lives inside the home	66.2% (86/130)
	The patient plays with the pet daily	74.6% (97/130)
	The pet eats in the kitchen with the family	37.6% (49/130)
	The patient and the pet share a bed, and/or the patient kisses/lets the pet lick his/her face	46.1% (60/130)
	The patient is directly involved in feeding the pet	40.8% (53/130)
	The patient is directly involved in the pet's hygiene	20% (26/130)
	The patient collects the animal's feces	15.4% (20/130)
	The patient cleans the animal's cage/basket/aquarium/terrarium	9.2% (12/130)

Animal feeding	Exclusively commercial processed food and/or home cooked food	93% (121/130)
	**Unprocessed and/or uncooked food**	**3.1% (4/130)** ^*∗*^

Hygiene (dogs/cats) (106/130)	1 time/week	6.6% (7/106)
	1-2 times/month	44.3% (47/106)
	Every 2–4 months	34.9% (37/106)
	Never	14.1% (15/106)

Veterinarian visits (dogs/cats)	≥3 times/year	38.7% (41/106)
	2 times/year	17.9% (19/106)
	1 time/year	22.6% (24/106)
	**<1 time/year**	**20.7% (22/106)** ^*∗*^

Vaccination up to date (dogs/cats)	Yes	94.3% (100/106)
	**No**	**5.7% (6/106)** ^*∗*^

Internal deworming (dogs/cats)	Monthly	11.3% (12/106)
	**Every 3 months or less**	**51.9% (55/106)** ^*∗*^
	**Less than once every 3 months**	**32% (34/106)** ^*∗*^
	**Never**	**4.7% (5/106)** ^*∗*^

^*∗*^Patients who did not comply with the veterinary recommendations for the general population and/or the recommendations of the guidelines for immunocompromised patients with pets (discouraged species or insufficient veterinary care). Deworming is generally recommended 4 times per year in outdoor cats and dogs although the recommended frequency can go up to once a month for immunocompromised patients. Bold values indicate patients who did not comply with the veterinary recommendations for the general population and/or the recommendations of the guidelines for immunocompromised patients with pets (discouraged species or insufficient veterinary care).

**Table 3:** Compliance with veterinary recommendations in dog and cat owners who completed the survey according to the patient's medical condition, age, treatments received, and type of pet.

Degrees of compliance with recommendations	Good compliance	Average compliance	Poor compliance	*p*
Type of medical conditions				
SOT	57% (38/67)	19% (13/67)	24% (16/67)	0.16
HSCT	46% (12/26)	35% (9/26)	19% (5/26)	
IEI	23% (3/13)	38% (5/13)	38% (5/13)	
Patient's age				
≤12 years	50% (23/46)	26% (12/46)	24% (11/46)	0.99
>12 years	50% (23/46)	25% (15/60)	25% (15/60)	
Patients under immunosuppressive therapy				
Yes	55% (37/67)	21% (14/67)	24% (16/67)	0.42
No	47% (14/30)	33% (10/30)	20% (6/30)	
Patients receiving IRT and/or antibiotic prophylaxis				
Yes	40% (6/15)	27% (4/15)	33% (5/15)	0.63
No	52% (47/91)	25% (23/91)	23% (21/91)	
Types of pet				
Dog	55% (41/75)	25% (19/75)	20% (15/75)	0.5
Cat	37% (7/19)	26% (5/19)	37% (7/19)	
Dog and cat	42% (5/12)	25% (3/12)	33% (4/12)	
Total	50% (53/106)	25% (27/106)	25% (26/106)	

^*∗*^Adequate compliance with veterinary recommendations was considered if the owners followed all the following recommendations: visiting the veterinarian at least once a year, complying with the vaccination schedule (core-vaccines), avoiding feeding the pet with unprocessed or raw food, and deworming the animal at least every 3 months. According to the number of measures with adequate compliance, we considered the following: (i) Good compliance: adequate compliance with the 4 items(ii) Average compliance: noncompliance in at least one of the items(iii) Poor compliance: noncompliance in at least two of the items For the statistical analysis, the dependent variable was the “compliance with the measures” and the independent variables were age, underlying condition, treatments received, and type of pet (dog, cat, or both).

## Data Availability

Most of the data used to support the findings of this study are included within the article. Other data used to support the findings of this study are available from the corresponding author upon request.

## Supplementary Materials

Supplementary Materials Survey of habits and risk behaviors for zoonosis acquisition in transplanted pediatric patients owning pets. The survey is divided into 4 sections, and the following data are collected: sociodemographic data, data related to the patient's disease and its treatment, data related to the presence of pets at home, and data related to healthcare professionals and recommendations given regarding pet ownership.
