# Supplementary material for: Immunocompromised Children and Young Patients Living with Pets: Gaps in Knowledge to Avoid Zoonosis
Source: Transbound Emerg Dis. 2023 May 31;2023:2151761. doi: 10.1155/2023/2151761 (PMC12017095; doi:10.1155/2023/2151761)
Supplement: Supplementary Materials — Survey of habits and risk behaviors for zoonosis acquisition in transplanted pediatric patients owning pets. The survey is divided into 4 sections, and the following data are collected: sociodemographic data, data related to the patient's disease and its treatment, data related to the presence of pets at home, and data related to healthcare professionals and recommendations given regarding pet ownership. [file 2151761.f1.docx]

**SURVEY OF HABITS AND RISK BEHAVIORS FOR ZOONOSIS ACQUISITION IN TRANSPLANTED PEDIATRIC PATIENTS OWNING PETS**

**SOCIODEMOGRAPHIC DATA**

First name and last name (initials):

Medical record number:

Sex:

- Male
- Female

Date of birth: ___/___/___

Date survey was conducted: ___/___/___

**DATA RELATED TO THE PATIENT'S DISEASE AND ITS TREATMENT**

Type of transplantation:

- Intestinal transplantation
- Liver transplantation
- Renal transplantation
- Heart transplantation
- Lung transplantation
- Multivisceral transplantation
- Bone marrow transplantation
- None

Date of transplantation (if applicable): ___/___/___

In case of bone narrow transplantation, end date of immunosuppressive treatment: ___/___/___

Drugs currently receiving (indicate those applicable):

- Cyclosporine (Sandimmun®)
- Tacrolimus (Prograf®)
- Sirolimus (Rapamune®)
- Everolimus (Certican®)
- [Mycophenolate mofetil](https://pubmed.ncbi.nlm.nih.gov/17130793/) (Cell-cept®, Myfortic®)
- Azathioprine (Imurel®)
- Systemic corticosteroids (Prednisone, prednisolone…)
- Other (specify): ___________

**DATA RELATED TO THE PRESENCE OF PETS AT HOME**

Indicate your degree of agreement with the following statement: “The presence of pets at home is a benefit for transplanted or immunosuppressed people”.

- Strongly agree
- Agree
- Neither agree nor disagree
- Disagree
- Strongly disagree

Indicate your degree of agreement with the following statement: “The presence of pets at home poses a risk for transplanted or immunosuppressed people”.

- Strongly agree
- Agree
- Neither agree nor disagree
- Disagree
- Strongly disagree

Indicate which of the following statements is more in line with your opinion:

- The emotional benefit of owning a pet outweighs the potential risk involved
- The risk of owning a pet outweighs its potential emotional benefits
- I do not have a formed opinion about this

Did the patient live at home with a pet before transplantation or starting immunosuppressive treatment?

- Yes
- No

If you answered "yes" to the previous question, did the patient stop living with the pet because of the transplant or immunosuppressive treatment?

- Yes
- No

Does the patient currently have a pet?

- Yes
- No

If applicable, indicate the number of pets the patient currently owns:

- 1
- 2
- 3
- ≥4

If applicable, indicate the type of pet:

- Dog
- Cat
- Rabbit
- Turtle/reptile
- Bird
- Hamster/guinea pig
- Other (specify): ________

Did the patient acquire any pet after transplantation or initiation of immunosuppressive treatment?

- Yes
- No

Time living with a pet from transplantation/start of immunosuppressive treatment to the present:

- <1 year
- 1-5 years
- 6-10 years
- >10 years

Is your pet under veterinary control?

- Yes
- No

If you answered "yes" to the previous question, specify the frequency of visits to the veterinary clinic:

- Less than once a year
- Once a year
- Twice a year
- Three times a year
- More than three times a year

Do you vaccinate your pet(s)?

- Yes
- No

Does your pet receive intestinal deworming?

- Yes
- No

If you answered “yes” to the previous question, specify the frequency of intestinal deworming:

- Once a month
- Every two months
- Every three months
- Every six months
- Once a year
- Sporadically

Is your pet treated for external parasites (collars, pipettes…)?

- Yes
- No

Indicate the type of food your pet eats:

- Exclusively commercial processed food (dry food, cans…)
- Home cooked food
- Raw homemade frozen food
- Raw homemade food not frozen

How often do you bathe your pet?

- Once a week
- Every 2-3 weeks
- Once a month
- Every 2-3 months
- Every 4 months or more
- Never

Is the patient usually the person in charge of the pet's care?

- Yes
- No

What activities does the patient perform with his/her pet(s)?

- Plays
- Feeds the pet
- Grooms the pet
- Cleans the cage/basket/aquarium/terrarium
- Walks the pet
- Collects feces
- Visits the veterinarian
- None

Where does the pet stay most of the time?

- Patient's room
- Patient's home, but outside his/her room
- Outside the patient's home (garden, yard, etc.)

**DATA RELATED TO HEALTHCARE PROFESSIONALS**

Before transplantation/beginning of treatment, did your doctor ask you about the presence of pets in your home or about your regular contact with animals?

- Yes
- No

If you answered “No” to the previous question, did you ask your doctor about living with pets or having regular contact with animals?

- Yes
- No

After transplantation/beginning of treatment, did your doctor ask you about the presence of pets in your home or about your regular contact with animals?

- Yes
- No

If you answered “No” to the previous question, after transplantation/beginning of treatment, did you ask your doctor about living with pets or having regular contact with animals?

- Yes
- No

Has your doctor given you recommendations about living with pets or having contact with animals?

- Yes
- No

If you answered “Yes” to the previous question, specify if the doctor recommended:

- Detachment of the pet
- Consulting your veterinarian for advice
- Following some indications regarding contact with pets and their care
- Other (specify): __________________

Has the patient modified his/her relationship with the pet(s) after the doctor’s recommendations?

- Yes
- No

If you answered “Yes” to the previous question, indicate if the patient:

- Has stopped having a pet
- Has bought a pet
- Has kept his/her pet(s) and changed habits

In case of keeping the pet and changing habits, indicate the changes that have been made:

- Patient has reduced contact with pet(s)
- Pet’s hygiene has improved
- Veterinary control of the pet has increased
- Other (specify):______________
